# Observation of nuclear quantum effects and hydrogen bond symmetrisation in high pressure ice

**DOI:** 10.1038/s41467-018-05164-x

**Published:** 2018-07-17

**Authors:** Thomas Meier, Sylvain Petitgirard, Saiana Khandarkhaeva, Leonid Dubrovinsky

**Affiliations:** Bayerisches Geoinstitut, Bayreuth University, Universitätsstraße 30, 95447 Bayreuth, Germany

## Abstract

Hydrogen bond symmetrisations in H-bonded systems triggered by pressure-induced nuclear quantum effects (NQEs) is a long-known concept but experimental evidence in high-pressure ices has remained elusive with conventional methods. Theoretical works predicted quantum-mechanical tunneling of protons within water ices to occur at pressures above 30 GPa, and the H-bond symmetrisation transition to occur above 60 GPa. Here we used ^1^H-NMR on high-pressure ice up to 97 GPa, and demonstrate that NQEs govern the behavior of the hydrogen bonded protons in ice VII already at significantly lower pressures than previously expected. A pronounced tunneling mode was found to be present up to the highest pressures of 97 GPa, well into the stability field of ice X, where NQEs are not anticipated in a fully symmetrised H-bond network. We found two distinct transitions in the NMR shift data at about 20 GPa and 75 GPa attributed to the step-wise symmetrisation of the H-bond.

## Abstract (web summary)

Hydrogen atoms in water ices, under pressures at which they might exist in ocean exoplanets and icy moons, exhibit dynamics that are still poorly understood. Here, ^1^H-NMR experiments approaching the Mbar range shed light on the symmetrisation of hydrogen bonds preceding and accompanying the transformation of ice VII into ice X.

## Introduction

Water in its liquid and solid forms is ubiquitous in nature, being one of the most abundant molecule in the universe, and it is thought to be a prerequisite to sustain life in our solar system and beyond. Water is one the main constituent of ocean exoplanets and icy moons like Ganymede, Europa, Enceladus, and Titan with possible existence of deep high-pressure ice layers in their internal structure. The hydrosphere of these bodies could be up to 900 km thick in icy satellites and up to several thousand kilometers in Ocean exoplanets^1,2^. Understanding their internal structure and evolution is crucial to determine their potential habitability and for interpreting upcoming NASA Europa Clipper and ESA Juice space missions^3,4^.

**Fig. 1:**
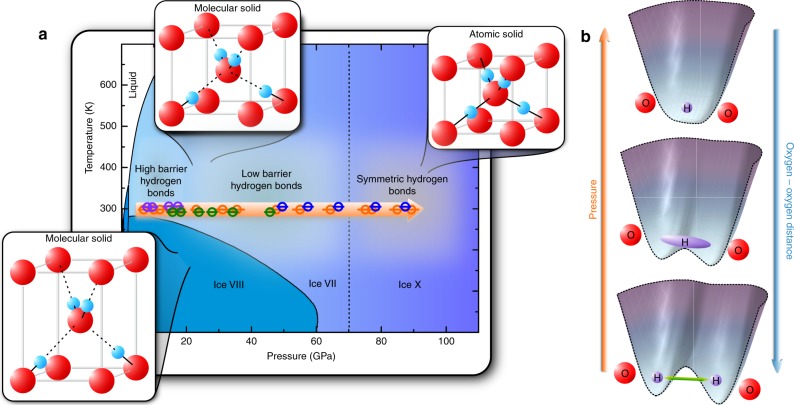
Phase diagram of H_2_O and schematic representation of hydrogen bond symmetrisation. **a** Phase diagram of H_2_O. The insets show the schematic symmetries of the high-pressure phases of ice VII, VIII, and X. Round symbols denote NMR measurements within this study, different colors relate different separate high pressure NMR experiments. **b** Sequence of hydrogen bond symmetrisation with pressure

Water molecules have been known for a long time to form a very specific type of chemical bonding—hydrogen bonds^5^. Under high pressure, the phase diagram of H_2_O exhibit an exotic behavior with more than 15 stable crystalline phases at variable temperature and pressure conditions^6^. The high-pressure region, above 3 GPa, is mostly dominated by the three ice phases VII, VIII, and X (Fig. 1). Both ice VII and VIII are molecular solids consisting of distinct H_2_O units linked to each other by hydrogen bonds. Ice X, on the other hand, exhibits a fully symmetrised hydrogen bond network, thus rendering a dissociation of the H_2_O molecules, forming an atomic solid at pressures of about 50–70 GPa at room temperature^7^. One of the most enigmatic phenomena in the high-pressure phase diagram of water is the transition from the hydrogen disordered phase ice VII into the hydrogen ordered phase of ice X^8^. It is widely believed that this transition is preceded by nuclear quantum effects (NQEs)^7,9^, or specifically, pronounced proton delocalization due to tunneling motion within the symmetric double-well potential of the hydrogen bonds in ice VII.

Evidence of hydrogen-bond symmetrisation and potential room-temperature proton tunneling in ice VII are sparse and often contradictory. This obviously relates to experimental difficulties. Hydrogen atoms remain effectively invisible to X-ray diffraction or emission spectroscopy^10,11^, leaving for observations only the heavier oxygen sublattice, which does not show significant transitions in the pressure range of interest^12,13^. While Raman-spectroscopy and neutron diffraction are more sensitive to H-bonds, they also yield ambiguous and often contradictory results,^14,15^.

Given these experimental difficulties, a direct observation of H-bond symmetrisation or NQEs remain mostly elusive in high-pressure experiments with the techniques discussed above.

One of the most promising spectroscopic methods to deal with these problems is nuclear magnetic resonance (NMR) spectroscopy, where proton NMR is best known for providing one of the highest possible NMR signal strengths, and allows direct observation of the electronic and structural environment of the nuclei. However, an application of NMR spectroscopy at high pressures, particularly in diamond anvil cells (DACs), was unfeasible, with set-ups unable to surpass 8 GPa on average, with rare exceptions reaching pressures above 10 GPa^16,17^.

Recently, a novel technique to detect the faint NMR signals by application of electro-magnetic Lenz lenses in Diamond indenter cells was introduced allowing for NMR experiments at pressures of up to 70 GPa^18^. In this study, we used a refined NMR resonator using a so called double stage Lenz lens (DSLL) structure^19^ at pressures approaching the megabar regime to investigate compression-induced nuclear quantum effects in ices. With this novel approach, we were able to follow the hydrogen bond symmetrisation in ice VII and its transition to the proton ordered phase X, one of the most sought after elusive effects in high-pressure sciences proposed 46 years ago.

## Results and Discussion

### High pressure NMR resonator

Recently, Spengler et al.^20^ demonstrated that the NMR sensitivity, in particular the limit of detection in the time domain, can be locally amplified with the use of so called Lenz lenses (LL). The LL-resonators form, in a general sense, a flux transformer picking up the high frequency B_1_ field generated by an excitation coil which is part of a standard LC tank circuit. The stored magnetic field energy will be deposited within a geometrically predefined area, leading to a locally enhanced B_1_ field. Recently, the significant advantage of these LLs for high pressure NMR was realized, and ^1^H-NMR spectra at pressures up to 70 GPa could be acquired in a toroidal diamond indenter cell^18^.

Here, a refined resonator structure has been employed which can be used within a standard DAC.

**Fig. 2:**
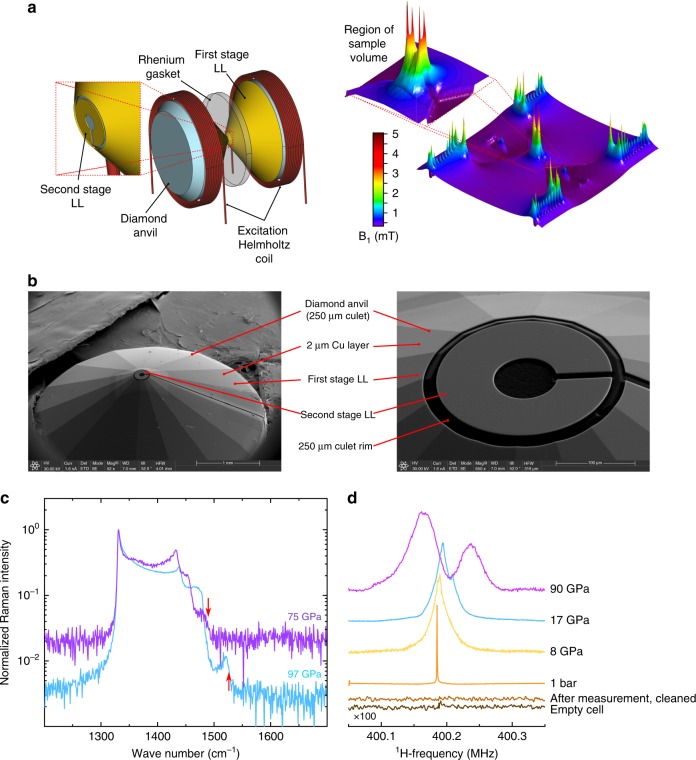
Summary of the experimental setup. **a** High-pressure NMR resonator setup. The inset shows the zoomed-in region around the anvil's culet. Both Lenz lenses are formed on the anvil's pavilion by copper deposition and subsequent shaping, using a focused ion beam. Simulations of the RF magnetic field generated by the resonator setup demonstrate the magnification in B_1_ at the sample cavity necessary for detecting NMR signals from the 100 pl sample cavity (80 µm diameter, 20 µm height prior to compression). **b** SEM images for the double stage Lenz lens (DSLL) resonator structure on one diamond. **c** Raman spectra accumulated at the diamond edge at the center of the sample cavity at pressures of 75 GPa and 97 GPa. Red arrows indicate the spectral position used for pressure determination. **d** Proton NMR spectra at different pressures as well as from empty cells, evidencing the origin of the acquired signals from the H_2_O samples from within the sample chamber

The basic idea is to accommodate a stable resonator structure for a pair of identical diamonds which can be driven by a high-inductance excitation coil. Figure 2a shows a schematic picture of such a setup. Numerical field simulations, right side of Fig. 2a, demonstrate that a DSLL arrangement is indeed able to significantly amplify the B_1_ field within the 100 pl sample chamber.

### High magnetic field data

**Fig. 3:**
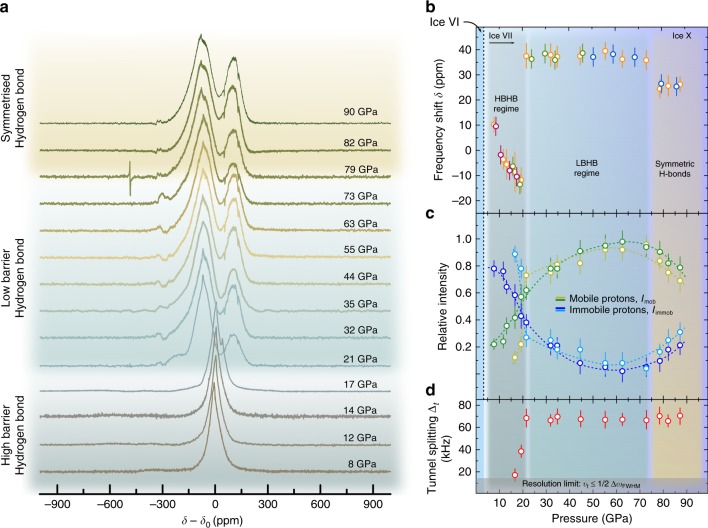
Summary of high-field data collected at 9.3 T. **a** Proton NMR signals of ice from 8 to 90 GPa. **b** chemical shifts relative to a water sample at ambient conditions. **c** Intensities from localized and tunneling protons. **d** tunnel splittings up to 90 GPa

^1^H-NMR spectra on ice from 8 to 90 GPa are shown in Fig. 3a. We performed several experiments using four independently loaded cells, with overlapping and reproducible results (Fig. 3b).

NQEs of protons have several observable effects on ^1^H-NMR spectra. First, random rapid tunneling from one to another minimum in a symmetric double-well energy potential will result in motional averaging of the NMR signals^21^. Second, ^1^H-NMR spectra in low barrier hydrogen bonds (LBHBs) exhibit significant de-shielding, with high proton shifts of about 20–40 ppm^22^.

Furthermore, it was shown that proton tunneling leads to a zero field splitting and detectable tunneling side bands. This effect was widely investigated for the tunnel rotation of methyl groups at low temperatures^23–25^. The simpler case of tunneling effects on NMR spectra when tunneling occurs solely along a linear axis, i.e., within a symmetric double-well potential, were predicted by Johnson^26^ and Johnston^27^. In general, rapid proton tunneling introduces an exchange between the allowed, magnetic transitions with Δ*m* = 1, and forbidden, or combination transitions. The position of the tunnel side bands (t.s.b.) greatly depends on the magnitude of the tunnel frequency with spectral positions at *υ* = *υ*_0_ ± *υ*_t_/2 where *υ*_0_ is the center frequency of the Δ*m* = 1 transition and *υ*_t_ is the tunnel frequency. Clough et al.^28^ showed that at low magnetic fields, the t.s.b. intensity is significantly improved, leading to a possible observation of higher order tunneling modes of up to 1 MHz.

**Fig. 4:**
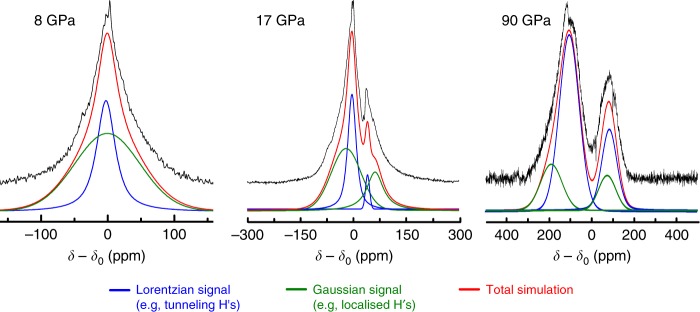
Results of the deconvolution of three ^1^H-NMR spectra (solid black lines) at pressures of 8, 17 and 90 GPa. Lorentzian (blue) and Gaussian (green) contributions as well as the resulting total simulation (red) are shown as well

The ^1^H-NMR spectra shown in Fig. 3a) were de-convoluted, see Fig. 4, into Gaussian and Lorentzian contributions and attributed to localized immobile protons and randomly tunneling protons delocalized over the energy hypersurface of the hydrogen bond. At pressures above 20 GPa, a pronounced tunnel splitting could be observed with tunnel frequencies between 70 and 80 kHz (Fig. 3d).

The proton signals of ice VII below 17 GPa could be best described with a superposition of two contributions of a Gaussian line of roughly 100 ppm in line width, and an almost purely Lorentzian signal of about 30 ppm width, see Fig. 3. While a hetero- and homonuclear dipole–dipole broadening can explain the Gaussian contribution, the sharp Lorentzian contribution is related to atomic or molecular motion of either single protons or whole-water molecules. However, it has been shown through molecular dynamic (MD) simulations^29^ and Raman spectroscopy^30^ that molecular diffusion in ice VII is negligible, leaving only single-protonic motion as a possible reason for such sharp proton signals in ice VII. Considering the geometry of the symmetric double-well potential of the HBHB regime, two possible mechanisms could originate protonic motion: thermally excited site hopping from one potential minimum to the other, or quantum-mechanical tunneling of the protons through the energy barrier. Theoretical analysis^31^ has shown that proton site hopping in ice VII at room temperature would be energetically unfavorable, leaving quantum-mechanical tunneling as the only possible effect responsible for the observed sharp signals.

Comparing the signal intensities of both Gaussian and Lorentzian contributions at 8 GPa, a ratio of 4:1 of protons localized in one of the two minima of the double-well potential, i.e., *I*_immob_, and rapidly tunneling protons, i.e., *I*_mob_, could be extracted. Using the same deconvolution procedure, the relative signal intensities of both contributions were analysed as a function of pressure and shown in the mid panel of Fig. 4b. For pressures up to 17 GPa, the relative intensity of the Gaussian contribution was found to continuously decrease; whereas, the Lorentzian contribution increased with pressure. At 17 GPa, the ^1^H-NMR signals exhibit a small splitting of about 18 kHz. This second signal was also de-convoluted using Gaussian and Lorentzian signal contributions. At about 18–22 GPa, a cross-over between *I*_immob_ and *I*_mob_ could be observed for both signals. At pressures of about 60 GPa, we found a maximum and minimum intensity of the Lorentzian and Gaussian contributions, respectively. At higher pressures, this trend reversed again with a second cross-over expected in a pressure of pressures of 100–120 GPa. This will occur when the majority of protons become localized in the fully symmetrised H-bonds of ice X.

The second proton signal observed at pressures above 17 GPa can be interpreted as result of tunnel splitting, and thus quantum-mechanical tunneling of protons within the hydrogen bond network. Another possible explanation would be due to the observed existence of multi-site disorder in ice VII^32^, but this can be ruled out considering that known proton chemical shift ranges of hydronium or hydroxyl ions are in the order of 10 ppm^33^, well within the observed linewidths of the spectra shown in Fig. 3a). Also, small variations in the O–H bond lengths, as inferred by neutron diffraction studies would lead to marginal increases in the Gaussian linewidths as the dipole–dipole interaction is slightly modified. Therefore, the presence of multi-site disorder would not be detected with this method as effects would most likely be below the spectral resolution limit or very small in magnitude.

**Fig. 5:**
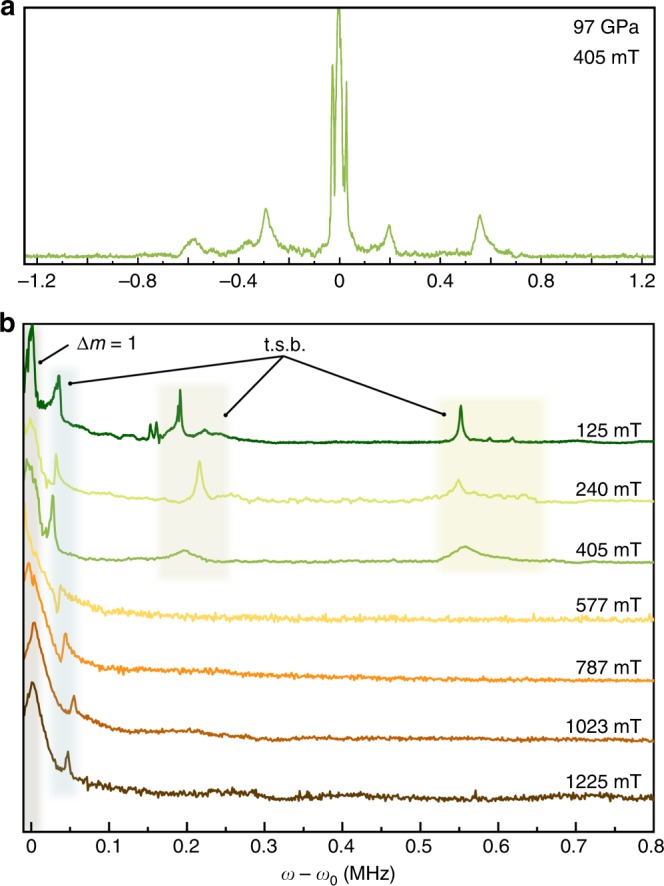
Low field NMR spectra of Ice X at 97 GPa. **a** Full ^1^H-NMR spectrum at a magnetic field of 405 mT, centered around the magnetic (Δ*m* = 1) signal. **b** High-field side of the ^1^H-NMR spectra between 125 and 1225 mT. Colored regions indicate the positions of the Δ*m* = 1 signals, as well as tunneling side bands (t.s.b.)

Another important aspect is the impact of non-hydrostatic pressure conditions on NMR spectra. In general, one might expect the most pronounced effects for quadrupolar nuclei (i.e., *I* > 1/2), such as the aluminium nucleus ^27^Al (*I* = 5/2). It could be shown^16^, that for these nuclei non-hydrostatic pressure conditions result in a significant line broadening originating from a non-isotropic deformation of the local charge distribution surrounding each quadrupole nucleus. However, *I* = 1/2 nuclei, such as hydrogen, do not possess a nuclear quadrupole moment which could interact with a potential electric field gradient influenced by non-hydrostatic pressure conditions, thus quadrupolar line broadening effects can be excluded^34^. Also, an another possible effect mediated through non-hydrostaticity would be a distribution of the diamagnetic shielding of the protons along a pressure gradient. In that case, the proton chemical shifts would vary depending on the respective pressure conditions. In principle, such an effect would lead to line broadenings which are in the order of the known chemical shift ranges of each nucleus. In the case of proton NMR, this would account to line broadening effects of about 10–20 ppm, which is much smaller compared to the observed linewidths of the spectra shown in Figs. 3–5. However, as the effects of non-hydrostaticity on NMR spectra and shifts have not yet been fully characterized in a series of similar compounds, these effects are unlikely to be the cause of the observed signals and shifts of the protons within the hydrogen bonds in ice VII or X.

Clough et al.^28^ argue that in the case of intermediate tunnel frequencies *υ*_T_ of 50–100 kHz, tunnel side bands, can be observed in high-field NMR. The intensity of these side bands fall off as *υ*_T_^−2^, thus only relatively small values of *υ*_T_ can be observed. Extractable tunnel splittings Δ_t_ from spectra shown in Fig. 3a) begin to appear at 17 GPa, and have been found to increase up to 75 kHz (Fig. 3d). Clearly, the resolution of this method is limited by the FWHM line width of the main NMR signal, as a splitting of Δ_T_ < 15 kHz would strongly overlap with the allowed magnetic (Δ*m* = 1) signal. Remarkably, *υ*_T_ does not change significantly between 20 and 90 GPa, in a pressure region where quantum-mechanical tunneling is believed to be absent due to the unimodal probability distribution of the protons localized in symmetric hydrogen bonds^31^. A possible reason for the constancy of *υ*_T_ over such a broad pressure range could be that several tunneling modes are present at significantly higher frequencies, remaining undetectable with high-field NMR. In this case, the observed Δ_T_ = 75 kHz (at pressures above 20 GPa) could be due to the lowest observable tunneling mode which corresponds to very small energy barriers.

### Low magnetic field data

In order to elucidate this issue, additional measurements at low magnetic fields have been conducted. Figure 5 shows several ^1^H-NMR spectra of ice X at 97 GPa at magnetic fields ranging from 125 to 1225 mT. As can be seen, in contrast to the high field spectra shown in Fig. 3a), the magnetic Δ*m* = 1 signal is flanked on both sides by tunnel side bands with tunnel splittings of about 40 kHz. The reason for the non-detectability of the down-field side band at 9 T is most likely due to significant broadenings and distortions of the forbidden transitions at these fields. Obviously, the general spectral positions of these inner tunnel side bands do not change relative to the magnetic signals, even after sweeping over a whole order of magnitude in B_0_. This zero field splitting can be considered a further evidence for pronounced proton tunneling. Strikingly, higher order tunneling side bands at about 200 and 560 kHz could also be observed, which indicates that the observed tunnel splitting at high field is indeed due to a lower lying tunneling mode.

## Discussion

The observed evolution of both *I*_immob_ and *I*_mob_ is consistent with the predicted evolution^31^ of the energy barrier of the double-well potential of the H-bond (Fig. 1b), i.e., increasing pressure reduces height and width of the barrier, increasing the probability of the protons to tunnel between the energy minima of the double-well potential. Thus, the relative intensity of signal due to localized protons declines; whereas, the intensity of signal originating from quantum-mechanical tunneling increases with decreasing oxygen–oxygen distances. At about 60 GPa, the majority of the protons participate in collective tunneling motion as *I*_mob_ reaches its maximum. At pressures above 60–70 GPa, the tunnel probability declines as height and width of the energy barrier approach zero, thus localizing the protons with pressure, leading to an increase in *I*_immob_.

The upper panel of Fig. 4b shows chemical shift values *δ* as a function of pressure. Two distinct transitions are evident at pressures of 20 and 75 GPa, respectively. While the pressure of the second transition is in very good agreement with the proposed transition for a symmetric hydrogen bond network in ice X, the first transition has not been observed with other methods and can be associated with a transition from the HBHB regime to the LBHB regime^35^. Thus, our NMR shift data indicates that a Low-Barrier Hydrogen Bond exists in ice VII not only at significantly lower pressures, but also in a pressure range where NQEs should be absent.

Our study indicates a much more complex scenario of the interplay between pressure-induced NQEs and the hydrogen bond symmetrisation in high-pressure ices than what could be anticipated from other experimental work in this field. In fact, up to this point no clear experimental distinction between the HBHB and LBHB regimes could be defined, whereas theoretical estimates vary often by tens of GPa between 30 and 60 GPa. Moreover, it could be shown that the LBHB–>SHB transition is indeed not a continuous one but exhibits a clear transition pressure of about 75 GPa.

## Methods

### Double stage Lenz lenz (DSLL) resonator preparation

The resonators were built in the following way. After pre-indenting a 200 µm rhenium gasket to about 15–20 µm, a thin layer of copper (1–2 µm) was deposited on the diamonds. The shape of the LLs was cut out from the copper layer using a focused ion beam (Scios Dual beam from FEI), Fig. 2b. As a result, the first stage LL typically runs from the outer rim of the diamonds pavilion toward close to the rim of the diamonds culet, with a thin 15 µm slit running all along the 1 mm pavilion (Fig. 2b). The second stage LL, is typically placed on the culet face having about 230 µm outer diameter and 80 µm inner diameter which closely follows the geometry of the gasket hole.

To ensure electrical insulation between both LLs and the metallic gasket, a 1 µm layer of Al_2_O_3_ was deposited on the gaskets.

### Preparation of the excitation coil

The excitation coil was made from 100 µm thick, PTFE insulated copper wire, consisting of 4 turns per coil, having a diameter of about 4 mm. After loading and closing of the cells, both coils were connected accordingly to form a Helmholtz coil pair yielding ~300 nH inductance. The overall resistance at 400 MHz was found to be about 1.5 Ω, thus the resonators quality should be 500. Using a spectrum analyzer, we found a quality factor of 530 in good agreement with the estimate.

### Test measurements at ambient pressure

First test measurements at ambient conditions, see ^1^H-NMR spectrum at 1 bar in Fig. 2d, demonstrates the excellent sensitivity of the DSLL setup. In order to prove that the recorded signals stem from the sample and not from spurious signals, additional measurements on an empty cell as well as on a recovered, opened and cleaned cell have been conducted, Fig. 2d. No significant proton NMR signals could be acquired in these cases.

### Pressure calibration

Pressure was measured using the first derivative of the pressure dependent shift of the first order Raman spectra of the diamond collected at the diamond edge in the center of the culet^36,37^, Fig. 2c shows two typical Raman spectra from two different DACs at pressures of 75 GPa and 97 GPa.

### High field NMR

High field NMR measurements have been conducted at a magnetic field of 9.03 T corresponding to a resonance frequency of about 400 MHz. Proton signals were collected using a *π*/2–*π*/2 solid echo pulse sequence with pulse separations of 50 µs in order to acquire the full spin echo. Typical r.f. pulses of 2 µs at 10 W average pulse power were used. Pressure dependent NMR shift measurements were calibrated against water at ambient conditions placed in a similar setup in a DAC, Fig. 2d, to account for intrinsic frequency shifts originating from the pressure cell assembly. The shift measurements were repeated with four different DACs at overlapping pressure ranges to ensure high reproducibility of the found effects.

### Low field NMR

Low field NMR measurements have been conducted in a DAC pressurized to 97 GPa, using a tunable electro-magnet of maximum 1.4 T field strength. The preparation procedure of this cell closely followed the above mentioned description. Proton signals have been accumulated between 125 and 1225 mT, using a single r.f. pulse of 1 µs length at an average pulse power of 35 W.

### Data availability

The data that support the findings of this study are available from the corresponding author upon reasonable request.
